# A Heart Rate Monitoring App (FibriCheck) for Atrial Fibrillation in General Practice: Pilot Usability Study

**DOI:** 10.2196/24461

**Published:** 2021-04-07

**Authors:** Simon Gabriël Beerten, Tine Proesmans, Bert Vaes

**Affiliations:** 1 Department of Public Health and Primary Care KU Leuven Leuven Belgium; 2 Qompium Hasselt Belgium

**Keywords:** atrial fibrillation, smartphone app, FibriCheck, primary care

## Abstract

**Background:**

Atrial fibrillation (AF) is a major risk factor for stroke. The current opportunistic screening procedure consists of pulse palpation and an electrocardiogram when an irregular rhythm is found. Smartphone apps that measure heart rhythm could be useful in increasing the detection of AF in a primary care setting.

**Objective:**

We conducted a pilot study with the smartphone app FibriCheck to assess whether the introduction of such an app is feasible.

**Methods:**

Four general practices across Flanders provided patient data for the study. Inclusion criteria for participants were aged 65 or older and a CHARGE-AF score of at least 10%. We excluded patients with known AF or a pacemaker. Participants were asked to measure at least twice a day with FibriCheck (for at least 14 days). They were provided the 36-Item Short Form Survey (SF-36) questionnaire both before and after the study, as well as different surveys concerning their user experience and general perception of technology.

**Results:**

There were 92 participants (36 women and 56 men). The study population was relatively homogenous concerning risk factors and medication use at baseline. During the study period, 5/86 (6%) participants were found to have AF (6 dropouts). The average study period was 23 days and the average number of measurements per day was 2.1. Patient compliance was variable, but high. On the whole, there were no appreciable changes in quality of life. The overall user experience and satisfaction were very high.

**Conclusions:**

FibriCheck is a relatively easy-to-use smartphone app to complement AF screening in primary care. Its implementation in this setting is certainly achievable, and one can expect high rates of patient compliance. Based on these results, a planned cluster randomized trial will be going ahead.

**Trial Registration:**

ClinicalTrials.gov NCT03509493; https://clinicaltrials.gov/ct2/show/NCT03509493

## Introduction

Atrial fibrillation (AF) has long been known as an independent risk factor for stroke [1]. It is highly prevalent among older patients in primary care, and the incidence is seemingly on the rise [2,3]. Hospitalizations for stroke are an important financial burden to the society, so a strategy for early and cost-effective screening interventions seems useful [4]. Current best clinical practice points to an opportunistic screening approach to detect AF, in which at-risk patients undergo routine pulse palpations and electrocardiograms (ECGs) when an irregular rhythm has been found [5,6]. Screening appears to be most cost-effective when done from age 65 [7]. Taking an ECG in routine general practice, however, is quite time-consuming. Furthermore, given the sometimes paroxysmal nature of AF, it could be missed by an opportunistic screening method during a routine consultation [8]. A significant proportion of these patients remain undiagnosed [9]. Holter measurements and event recorders could partially remedy this issue, but interpretation is again very time-consuming and unlikely to be very cost-effective [10].

Portable heart rate monitoring devices have recently been introduced to provide an on-the-go way to check for arrhythmias in general practice [11]. They could provide a convenient add-on to current opportunistic screening. Since the advent of smartphones, efforts have been made to use the phone’s built-in camera to register heart rhythm via photoplethysmography [11,12]. Today, various smartphone apps are available [11,13,14], and they all perform well in terms of diagnostic accuracy and yield [15] and appear to be cost-effective [7].

In Belgium, the Hasselt-based firm Qompium has developed one such smartphone app named FibriCheck. The diagnostic accuracy of this app has already been studied previously [16]. An upcoming cluster randomized controlled trial will study its efficacy as a diagnostic tool to facilitate screening. This pilot study aimed to assess the ease of use and implementation of the FibriCheck app in a primary care setting.

## Methods

### Recruitment

#### Practices

This feasibility study ran from June to December 2017. Four general practices across Flanders were recruited in this study (Table 1). Every practice used different medical software to code diagnoses and parameters: a deliberate choice to test the ease with which data could be derived from each software package. Each practice was asked to include around 20 patients.

**Table 1:** Overview of recruiting practices.

Name of the practice	Place	Team	Medical software
Huisartsencentrum Millegem	Mol, Antwerp	4 GPs^a^	MediDoc
Groepspraktijk Hoeilaart	Hoeilaart, Flemish Brabant	6 GPs, 2 GP trainees	CareConnect
Huisartsenpraktijk Keerbergen	Keerbergen, Flemish Brabant	2 GPs, 1 GP trainees	Windoc
Praktijk Gilissen	Riemst, Limburg	2 GPs, 1 GP trainees	Prodoc

^a^GP: general practitioner.

#### Participants

We opted to only include patients at high risk for AF to test the suitability of the FibriCheck app. We only included the older population, as AF is more prevalent in this group and they would therefore benefit the most from this intervention. There was no control group. The 5-year risk of AF is commonly indicated using the CHARGE-AF score [17]. This score was calculated manually for each potential participant, by extracting the necessary data from the patient file. Frailty score was calculated according to Tocchi et al [18]. A score of 0 means no frailty, a score of 1-3 means increased risk of frailty, and a score of 4 or more indicates definite frailty.

We opted to exclude patients already on anticoagulants (the mainstay of treatment for AF with higher cardiovascular risk), as this screening intervention would probably not lead to any relevant change in treatment for that population.

The inclusion criteria were (1) a CHARGE-AF score of 10% or more and (2) aged 65 or older. Exclusion criteria were (1) known or already diagnosed AF (including known paroxysmal AF), (2) patient has a pacemaker, (3) patient takes oral anticoagulants, and (4) patient is unable to use a smartphone app due to physical, visual, or cognitive impairment.

Upon selection, participants were asked to sign a consent form to be included in the study.

Participants were given a unique coded account number, which is linked to the specific FibriCheck app on their smartphone. Smartphones were provided in case participants did not own one themselves; others just had to install the app.

At recruitment and at the end of the study, an ECG and a FibriCheck measurement were taken.

The time the physician spent to explain the study to the participants and on educating them how to use the FibriCheck app was registered.

In addition, physicians were asked to indicate what they would have done if the FibriCheck app was not available (ie, if they were not included in the study). There were 2 options: no action (and wait for the patients to consult on their own) or advise a follow-up consultation. Physicians could also indicate what exactly they would have done without the availability of the app, and how many consultations they normally anticipated during a 1-year period.

#### Measurements

The FibriCheck app uses the smartphone’s built-in camera to measure the heart rate. The users are instructed to place their finger on the camera and wait for 60 seconds as a measurement is taken, preferably in the seated position with the arm resting.

At the start of the study, participants were asked to measure at least twice a day with the FibriCheck app and to indicate if they experienced any symptoms preceding the measurement. The minimum required measuring period was 2 weeks; the individual participant’s study period ended after a maximum of 4 weeks. Patients were considered incompliant and excluded when there were no measurements for 2 weeks or more during the study period, or if they were lost to follow-up.

After measuring, the FibriCheck app reviewed every measurement immediately afterward, with 4 possible outcomes: normal measurement (no tachycardia, no extrasystole, no irregular rhythm; indicated in green), inadequate signal (indicated in blue), measurement requiring urgent attention (possible AF; not signaled to participants in the context of this pilot study), and warning (usually more than 4 extrasystoles, or bradycardia or tachycardia: indicated in orange). Participants were also able to indicate their stress levels with each measurement. The score ranged from 0 (low stress) to 10 (highest stress) with step intervals of 2.5.

#### Quality of Life

Patients were asked to fill in the 36-Item Short Form Survey (SF-36) questionnaire, to get an impression of their quality of life at baseline. This survey contains 36 questions from various health domains, such as physical functioning or emotional health [19]. It uses graded responses: answers corresponding to more favorable health states receive higher scores (the minimum and maximum being 0 and 100, respectively). The scores for the questions on a specific health domain are then averaged to compile a subtotal score. Each participant was asked to complete the questionnaire again at the end of the study period. The SF-36 questionnaire has been validated for use in general practice [20]. We used the Dutch translation in this study [21].

#### Technology Perception—App Usability

Participants were provided questionnaires at the beginning of the study, concerning their perception and familiarity of current technology. At the end of the study, a general questionnaire about their use of the FibriCheck app was provided. Answers were graded on a 5-step scale, ranging from “completely disagree” to “fully agree.” These questionnaires were created by the authors and were not validated previously.

### Statistical Analysis

A 2-proportion, 2-tailed *Z* test was used to analyze the difference in risk factors and medication between male and female participants in the study. To compare the scores on the various domains of the SF-36 questionnaire, we used a 2-tailed Wilcoxon signed-rank test, as we did not assume normality. The value for any missing item was imputed as the mean value for nonmissing items.

We refrained from calculating the total average score of the SF-36 questionnaire. This is often done to form an idea about the general health of study participants. However, this supposes a 50/50 equilibrium between the mental and physical aspects of health, and this practice is generally discouraged [22].

The minimal clinically important difference for the health-related SF-36 questionnaire was calculated according to earlier studies for similar populations [23,24]. We used a distribution-based method, as an anchor-based method was not feasible for this pilot study. A cut-off value of 1 standard error of measurement (SEM) was used to define a meaningful improvement or deterioration, in line with previous studies [24].

The following formula was used to calculate the SEM, with *σ* being the SD of a particular test and *r* the reliability coefficient or Cronbach alpha of the same test [23]:

*σ*√(1–*r*)

Data for this formula, as applicable to the SF-36 questionnaire, were gathered from the Medical Outcomes Study [25].

The answers to the questionnaires concerning technology perception and usability of the FibriCheck app were weighted according to importance: for instance, “completely agree” (or a similar answer) was assigned a value of 2, “agree” a value of 1, “completely disagree” a value of –2, and so on.

For other data, we used descriptive statistics throughout.

### Ethics Approval

This pilot study was approved by the Ethical Committee of Hospital Zuid-Oost Limburg (Genk, Belgium) on June 6, 2017 (registration number B371201731704).

## Results

### Participants

A total of 92 participants were recruited from the 4 practices listed in Table 1: (1) Huisartsencentrum Millegem (17 patients), (2) Groepspraktijk Hoeilaart (35 patients), (3) Huisartsenpraktijk Keerbergen (20 patients), and (4) Praktijk Gilissen (20 patients).

There were 36 female and 56 male participants in the study population. The mean age was 78 (SD 8.1; range 45-94 years). Ultimately, we did include 4 patients under 65 who met the other inclusion criteria. The rest of the population was 65 or older. The mean BMI was 28.1 kg/m^2^ (SD 4.6; range 17.3-42.7 kg/m^2^). In our study population, the mean frailty score was 2.6 with 21/92 participants (23%) having a score of 4 or more.

Before the study commenced, participants were asked whether they had used a smartphone before or had the ability to use a smartphone correctly. There was a response rate of 96% (88/92 participants). In total, 16% (14/88 participants) had a smartphone and knew how it worked, 32% (28/88 participants) did not have an idea of what to do with a smartphone, 13% (11/88 participants) had used a smartphone before and could manage, while 40% (35/88 participants) had used a smartphone but found they needed help.

At recruitment and at the end of the study, an ECG and a FibriCheck measurement were taken. Before commencement, 93% of participants (86/92) were in sinus rhythm, 6% (5/92) had ectopic beats, and 1% (1/92) had atrial flutter.

On average, physicians spent around 15 minutes to get participants started with the smartphone and the app (range 5-40 minutes), and another 21 minutes to fill in the necessary administrative paperwork (consent forms, patient education leaflets, etc.; range 10-40 minutes).

The risk factors of the participants, as well as the different medications they were taking at baseline, are listed in Multimedia Appendix 1.

Physicians would have chosen the “wait and see” approach for 67/92 patients (73%), if the FibriCheck app would not have been available. In this scenario, they anticipated on average between 2 and 3 consultations over a 1-year period. The mean CHARGE-AF score for participants in the “follow-up” group was 20.89, whereas that in the “wait and see” group was 18.60.

### Measurements

There were 24 consultations with 18/92 patients (20%) purely because of a FibriCheck finding, as well as 3 hospital admissions (3%) indirectly resulting from a finding on the app. All the aberrant rhythms detected during and at the end of the study are summarized in Table 2.

**Figure 1:**
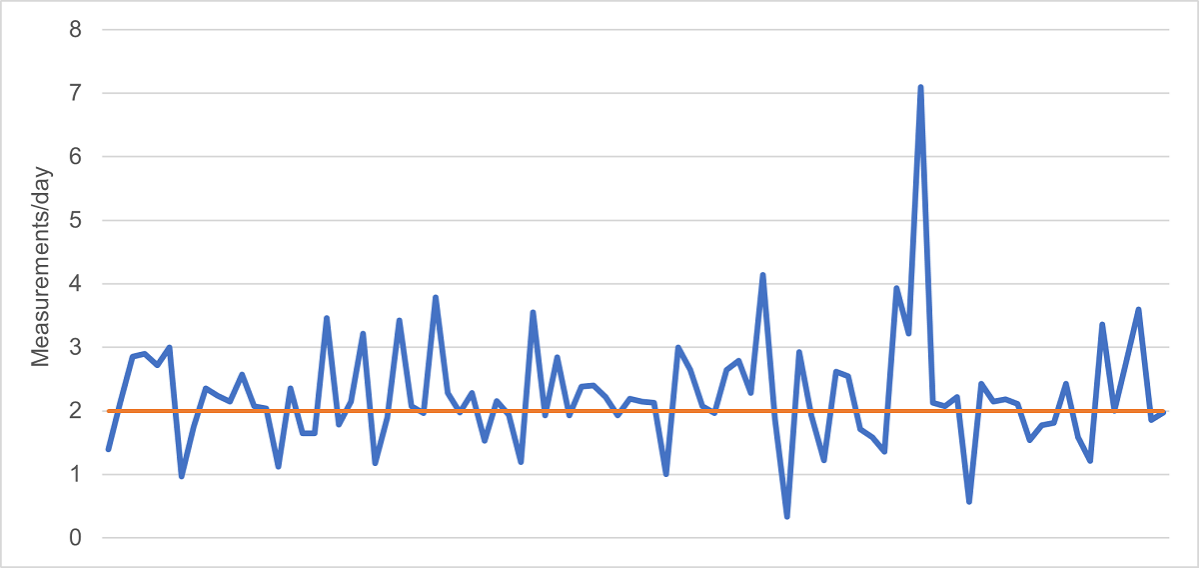
Average number of measurements per day, per participant.

The average participant study period was 23 days. Participants conducted an average of 49.5 measurements during that time, which amounts to 2.1 measurements per day. Figure 1 shows the average number of measurements for each participant as a line. The “2 measurements per day” criterion is highlighted, which gives an idea about participant compliance.

**Table 2:** Rhythms detected during the study (N=86).^a^

Heart rhythm	Value, n (%)
Ectopic beats (SVES^b^/VES^c^)	10 (12)
Tachycardia	2 (2)
Atrial fibrillation	5 (6)

^a^Six dropouts: 4 exclusions by patient request, 2 excluded due to incompliance.

^b^SVES: supraventricular extrasystoles.

^c^VES: ventricular extrasystoles.

**Figure 2:**
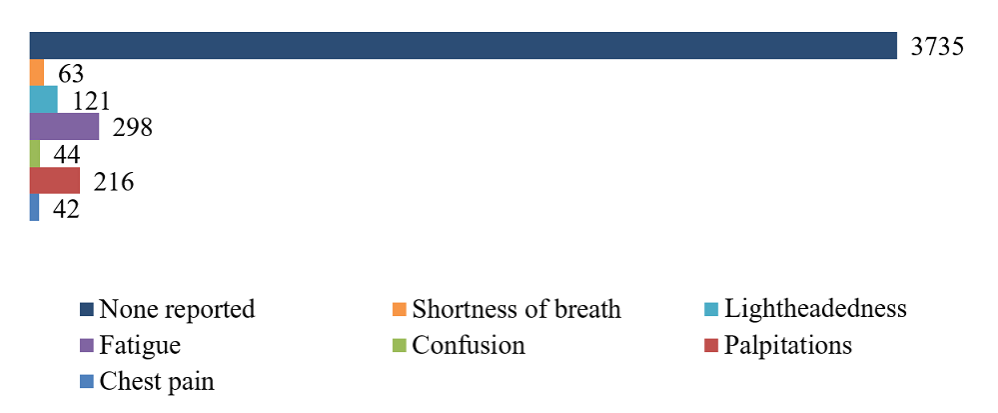
Proportion of reported symptoms (N=4449); 40 measurements were not validated by the algorithm due to connection errors.

**Figure 3:**
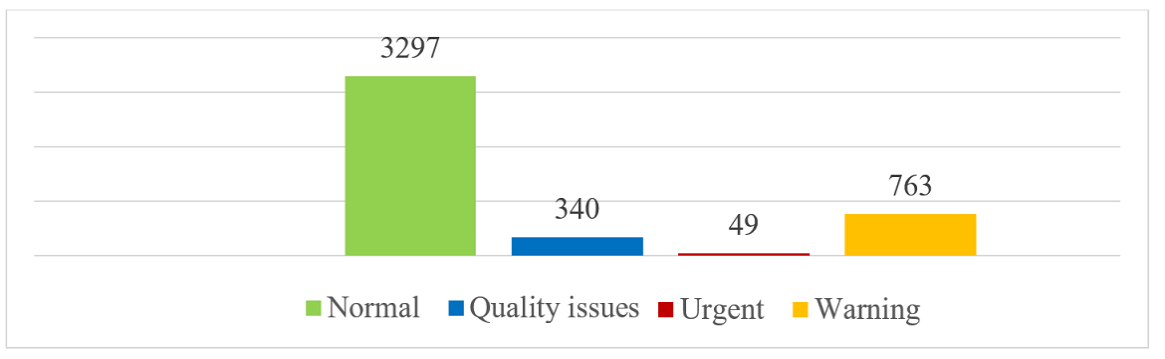
Number of different FibriCheck measurements (N=4449); 40 measurements were not validated by the algorithm due to connection errors.

A total of 4489 validated measurements were taken for 90 participants in total. Approximately 71% of participants (64/90) had 2 or more measurements per day. A summary of the most common symptoms accompanying the measurements is displayed in Figures 2 and 3. Most measurements did not report a symptom and for each measurement multiple symptoms could be reported. There were a total of 3313 measurements that had a stress level registered. The mean stress level was 2.29.

### Quality of Life

In total, 54 participants filled in the SF-36 questionnaire at the start of the study. We excluded 4 patients from the final analysis, because they failed to complete the questionnaire at the end of the study. Multimedia Appendix 2 shows the results for the 50 participants who completed the questionnaire.

### Technology Perception and App Usability

The data on the technology perception, gathered at the beginning of the study, and the data on the use of the FibriCheck app, are summarized in Multimedia Appendix 3. The specific questions belonging to each category, together with their weighting, can be found in Multimedia Appendices 4 and 5. Results are weighted according to the different response categories: more positive or more negative responses are thus weighted accordingly. For purposes of readability, we did not plot the neutral answers. The response rates for the technology and FibriCheck surveys were 89% (82/92 participants) and 77% (71/92 participants), respectively.

Participants were most satisfied with the following aspects of the app: simplicity (52/71, 73%), on-the-go heart rhythm analysis (52/71, 73%), and the possibility to be followed remotely (48/71, 68%).

## Discussion

### Principal Findings

This pilot study concerned the ease of use and implementation of the AF case-finding app FibriCheck in primary care. The study population was rather homogenous, and smartphone familiarity at baseline was relatively poor. We found a high measurement compliance, with most participants finding the app easy to use. AF was detected in 5/86 participants (6%). Overall, the user experience was positive, and most participants agreed the app gave them a feeling of reassurance and could benefit their doctor–patient relationship.

### Participants

This pilot study merely focused on the feasibility of the introduction of an AF case-finding app in a primary care setting. As we were testing the app itself rather than its effect on the detection rate of AF, we did not add a control group and focused only on those patients we thought would benefit the most from such an app.

The study population of 92 participants was predominantly male, but there were no differences between men and women regarding risk factors and medication use at baseline. However, the proportion of men with a history of thrombosis or peripheral vascular disease was significantly greater (*P*=.02 for both). In addition, our study population was generally overweight, so it might not be representative of the average general practice population.

Smartphone familiarity was rather poor in our study. Use of smartphones does tend to be lower in older populations, for reasons such as a lack of interest in current technologies, visual impairments, or financial problems [26]. However, most participants in our study had no problems using the FibriCheck app. There was some preliminary work involved in acquainting participants with the app, which took around 15 extra minutes in our study. This is not extraordinary, though quite significant in the daily schedule of a general practitioner.

### Measurements

An important feature of this pilot study was patient compliance. As this was a preliminary study in anticipation of a larger cluster randomized trial, we set the bar for noncompliance quite low: only participants with no measuring activity for 2 weeks or more were excluded from the study.

The 2-week mark proved easy to reach: the average study period was 23 days. This timeframe was chosen deliberately, as 14 days seem to be a sufficient time to detect most AF cases [27]. The average number of measurements per day was 2.1, with a large spread (Figure 1). Around 71% (64/90) of participants measured twice a day or more. Methodologically similar studies found compliance rates ranging from 75% to 95% [13,27], whereas in another study the measurements were performed in the presence of a trained personnel [14].

The proportion of participants with AF in this study was 6% (5/86), higher than other comparable studies or the general population [13,27,28], most likely because we only included high-risk patients and possibly because of the effect of screening. Another device study [14] differed in the study design, which makes comparison difficult. The most indicated symptoms when conducting a FibriCheck measurement (if there were any) were fatigue and palpitations, in line with findings from a similar study [13].

### Quality of Life

We asked participants to complete the SF-36 questionnaire both before and after the study, to see if there were any appreciable changes in quality of life. There were no changes in any of the SF-36 health domains, except for Emotional Well-being, which showed a significant decrease (*P*<.001). This could be due to any number of factors: added stress due to the enrollment in the study in general, or anxiety because of preoccupation with heart disease in particular, possibly amplified by having to test the heart rhythm at least twice a day. Patient anxiety could potentially be diminished when the device does not give direct feedback about the results, as in an ECG app [13].

### Technology Perception and App Usability

The participants in our study were very accepting of the current technology and very open to try the FibriCheck app, as most could see its benefit if data protection was properly ensured. Another study found that technology acceptance among the elderly seems to be increasing, provided certain barriers (privacy issues and design) are well taken care of [29]. The FibriCheck app was found to be easy to use, and it gave most participants a feeling of reassurance and safety. They also believed it improved their doctor–patient relationship.

### Strengths and Limitations

This was the first study to assess the feasibility of integrating the FibriCheck app in a primary care setting. The study population was sufficiently homogenous to be able to draw some relevant conclusions. There was no control group, as this was merely a feasibility study. A comparison with routine care was thus not possible.

Overall, there were varying amounts of missing data, though generally not very much (up to 4.0% [26/644 data points] for the demographic data) and at acceptable levels for the different surveys. We decided to ignore missing data when reporting descriptive statistics but opted for mean value imputation in the surveys.

### Conclusions and Implications for Practice

Integrating a smartphone app such as FibriCheck in primary care seems to be an easy way to complement routine screening. We found high rates of patient satisfaction, reassurance, and compliance. Smartphone familiarity might still be an issue, although most participants of this study had no problem using the app.

The findings in this study pave the way for the routine use of new technology in a general practice setting. Given the widespread use of smartphones, screening apps could be a cost-efficient way of complementing routine care with smart technology. Apps like FibriCheck could potentially increase AF detection in general practice, compared with traditional screening, decreasing the burden of associated morbidity and mortality. As technology acceptance among the elderly will continue to increase, so will the relevance of screening apps. Cost–benefit issues and potential barriers to large-scale implementation have yet to be identified.

A cluster randomized trial, to compare the diagnostic yield of FibriCheck with usual care, is planned (ClinicalTrials.gov NCT04545723). In short, the results of this pilot study indicate that the implementation of a smartphone screening app in general practice is easy and feasible, which could guide future trials by shifting the focus away from practical issues, toward the specifics of screening and diagnosis.

## Appendix

Multimedia Appendix 1 Risk factors and medication of participants at baseline, for men and women.

Multimedia Appendix 2 Comparison of SF36 domain scores, at start and end of study (N=50).

Multimedia Appendix 3 Questionnaires concerning technology perception and FibriCheck usage.

Multimedia Appendix 4 Technology perception questionnaire with weighting.

Multimedia Appendix 5 FibriCheck usage questionnaire with weighting.

## Abbreviations

AF: atrial fibrillation
ECG: electrocardiogram
SEM: standard error of measurement
