# Supplementary material for: A Heart Rate Monitoring App (FibriCheck) for Atrial Fibrillation in General Practice: Pilot Usability Study
Source: JMIR Form Res. 2021 Apr 7;5(4):e24461. doi: 10.2196/24461 (PMC8060868; doi:10.2196/24461)
Supplement: Multimedia Appendix 1 [file formative_v5i4e24461_app1.pdf]

		Men (N=56), n (%)	Women (N=36), n (%)	*P* value [b]
**Risk factors**				
	Diabetes	23 (41)	9 (25)	.11
	Arterial hypertension	48 (86)	26 (72)	.11
	Heart failure	2 (4)	4 (11)	.15
	Stroke/TIA	5 (9)	5 (14)	.45
	Cardiac ablation	3 (5)	1 (3)	.56
	Other cardiac surgery	10 (18)	2 (6)	.09
	Cardioversion	0 (0)	1 (3)	.21
	Thrombosis (DVT/PE)	14 (25)	2 (6)	*.02*
	Hypercholesterolemia	23 (41)	14 (39)	.83
	Peripheral vascular disease	22 (39)	6 (17)	*.02*
	Obesity	15 (27)	8 (22)	.62
	Smoking	8 (14)	1 (3)	.07
**CHARGE-AF score**				
	0-10 (“low”)	16 (29)	15 (44)	.13
	11-20 (“medium”)	18 (32)	12 (35)	.76
	21-30 (“high”)	11 (20)	4 (12)	.33
	31+ (“very high”)	11 (20)	3 (9)	.17
**Medication**				
	Beta-blockers	26 (46)	14 (39)	.48
	ACE-I/ARBs	17 (30)	16 (44)	.17
	CCBs	8 (14)	6 (17)	.76
	Diuretics	10 (18)	6 (17)	.88
	No CV	16 (29)	13 (36)	.45
	1-2 CV	32 (57)	16 (44)	.23
	>2 CV	8 (14)	7 (19)	.52

[a] Abbreviations: TIA = transient ischemic attack, DVT = deep venous thrombosis, PE = pulmonary embolism, ACE-I = angiotensin-converting-enzyme inhibitors, ARB = angiotensin II receptor blockers, CCB = calcium channel blocker, CV = cardiovascular medication.

[b] Two-proportion, two-tailed *Z*-test; 95% confidence.
