# Supplementary material for: A Heart Rate Monitoring App (FibriCheck) for Atrial Fibrillation in General Practice: Pilot Usability Study
Source: JMIR Form Res. 2021 Apr 7;5(4):e24461. doi: 10.2196/24461 (PMC8060868; doi:10.2196/24461)
Supplement: Multimedia Appendix 2 [file formative_v5i4e24461_app2.pdf]

SF36 domains	Mean score at start, (SD)	Mean score at end, (SD)	MCID (= 1 SEM)	*P* value [c]
Emotional well-being	50.4 (9.6)	42.6 (6.9)	7.0	*<.001*
Energy/fatigue	51.1 (11.7)	51.0 (11.2)	8.4	.76
General health	49.5 (10.9)	48.4 (9.4)	10.0	.64
Pain	71.5 (23.3)	74.3 (23.9)	12.0	.35
Physical functioning	57.9 (24.2)	58.7 (25.2)	7.3	.79
Role limitations/emotional	77.0 (31.8)	84.9 (30.1)	16.8	.10
Role limitations/physical	69.4 (37.1)	73.4 (35)	16.3	.20
Social functioning	83.8 (17.4)	83.8 (20.5)	9.9	.96

[a] Abbreviations: SD = standard deviation, MCID = minimal clinically important difference, SEM = standard error of measurement.

[b] A higher value indicates a more favorable health status (minimum: 0, maximum: 100).

[c] Wilcoxon signed-rank test, two-tailed; 95% confidence.
