# Supplementary material for: A Heart Rate Monitoring App (FibriCheck) for Atrial Fibrillation in General Practice: Pilot Usability Study
Source: JMIR Form Res. 2021 Apr 7;5(4):e24461. doi: 10.2196/24461 (PMC8060868; doi:10.2196/24461)
Supplement: Multimedia Appendix 3 [file formative_v5i4e24461_app3.pdf]

**Technology perception**		
	Ease of smartphone usage	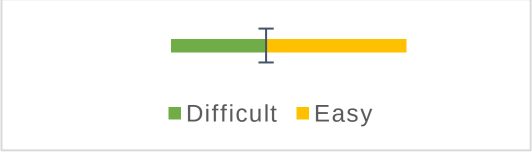<br>Neutral: 15%
	Smartphone accessibility	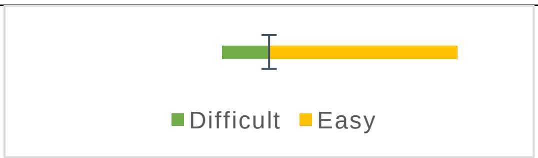
	Technology acceptance	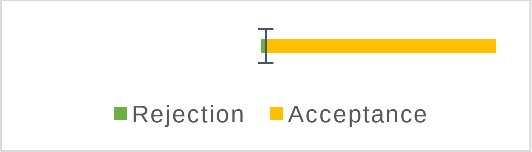<br>Neutral: 8%
	Data protection	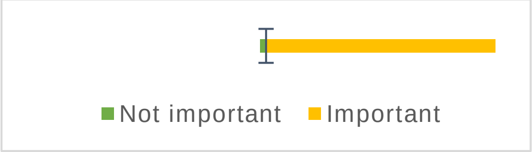<br>Neutral: 9%
**FibriCheck® usage**		
	Difficulty	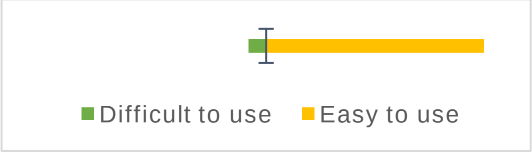<br>Neutral: 13%
	General satisfaction	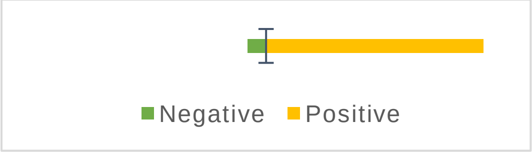<br>Neutral: 12%
	Feeling of safety / Reassurance	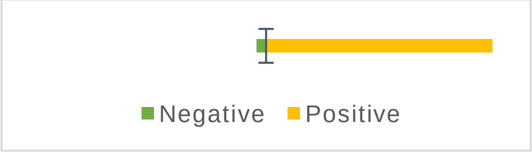<br>Neutral: 9%
	Physician relationship	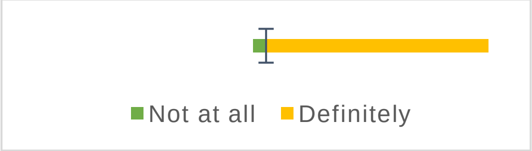<br>Neutral: 21%
