# Supplementary material for: A Heart Rate Monitoring App (FibriCheck) for Atrial Fibrillation in General Practice: Pilot Usability Study
Source: JMIR Form Res. 2021 Apr 7;5(4):e24461. doi: 10.2196/24461 (PMC8060868; doi:10.2196/24461)
Supplement: Multimedia Appendix 4 [file formative_v5i4e24461_app4.pdf]

Subject	Question	**Response categories**				
		Fully agree	Agree	Neutral	Disagree	Fully disagree
Ease of smartphone usage	I think I need help to install the application	+2	+1	0	-1	-2
	I had to train myself to use a smartphone for this project	-2	-1	0	+1	+2
			Yes		No	
	I worked with a smartphone before		+1		-1	
		Never used	Once	I can use it	Useful	Very useful
	How easily can you operate a smartphone?	-2	-1	0	+1	+2
			Yes		No	
Smartphone accessibility	I already have a smartphone		+1		-1	
	I have internet access		+1		-1	
		Yes		No internet		No children
	I have (grand)children who use a smartphone	+1		-1		0
		Fully agree	Agree	Neutral	Disagree	Fully disagree
Technology acceptance	Are you open for changes or new innovations?	+2	+1	0	-1	-2
	Do you trust that your smartphone can accurately register measurements?	+2	+1	0	-1	-2
	Healthcare should get more initiatives to follow patients with mobile technology	+2	+1	0	-1	-2
		Fully agree	Agree	Neutral	Disagree	Fully disagree
Data protection	Protection of personal information and privacy is important in general	+2	+1	0	-1	-2
	My privacy is important	+2	+1	0	-1	-2
