# Supplementary material for: A Heart Rate Monitoring App (FibriCheck) for Atrial Fibrillation in General Practice: Pilot Usability Study
Source: JMIR Form Res. 2021 Apr 7;5(4):e24461. doi: 10.2196/24461 (PMC8060868; doi:10.2196/24461)
Supplement: Multimedia Appendix 5 [file formative_v5i4e24461_app5.pdf]

Subject	Question	Response categories				
		Fully agree	Agree	Neutral	Disagree	Fully disagree
Difficulty of the app	I am convinced that other users have no problems with the application	+2	+1	0	-1	-2
	The application was easy to use	+2	+1	0	-1	-2
	I need help to conduct measurements	-2	-1	0	+1	+2
	The application is complex to use	-2	-1	0	+1	+2
	The different functions of the application were clear	+2	+1	0	-1	-2
	I can use the application independently	+2	+1	0	-1	-2
	The result screen was clear	+2	+1	0	-1	-2
		Fully agree	Agree	Neutral	Disagree	Fully disagree
General satisfaction	I was satisfied about the FibriCheck® product	+2	+1	0	-1	-2
	I would like to continue to use the application after this project	+2	+1	0	-1	-2
	FibriCheck® delivers an added value for the patient	+2	+1	0	-1	-2
	The application was not well designed	-2	-1	0	+1	+2
	The application was bad and not developed properly	-2	-1	0	+1	+2
	I found it positive to register my symptoms	+2	+1	0	-1	-2
		Fully agree	Agree	Neutral	Disagree	Fully disagree
Feeling of safety / Reassurance	FibriCheck® made me feel safe	+2	+1	0	-1	-2

	Did you feel safe using FibriCheck®?	+2	+1	0	-1	-2
	Do you feel reassured knowing that you can always receive support?	+2	+1	0	-1	-2
Feeling of safety / Reassurance	Do you feel reassured knowing that your physician can remotely follow your results?	+2	+1	0	-1	-2
	I find it positive that all my data is automatically sent to my healthcare provider	+2	+1	0	-1	-2

		Fully agree	Agree	Neutral	Disagree	Fully disagree
Physician relationship	I feel that FibriCheck® benefits the relation with my healthcare provider	+2	+1	0	-1	-2
	The communication with my healthcare provider has improved	+2	+1	0	-1	-2
	Do you expect that the communication with your healthcare provider will improve?	+2	+1	0	-1	-2
